# Supplementary material for: Enhancing speed of pinning synchronizability: low-degree nodes with high feedback gains
Source: Sci Rep. 2015 Dec 2;5:17459. doi: 10.1038/srep17459 (PMC4667188; doi:10.1038/srep17459)
Supplement: Supplementary Information [file srep17459-s1.pdf]

Supplementary Information

Enhancing speed of pinning synchronizability: low-degree nodes with high feedback gains

Ming-Yang Zhou, Zhao Zhuo, Hao Liao, Zhong-Qian Fu and Shi-Min Cai

I. SI NOTES: STABLE CONDITION

Given a network controlled by feedback, the state equations of the network are[1–3]

$$\dot{\mathbf{x}}_i = f(\mathbf{x}_i) + c\sum_{j=1}^{N} a_{ij}\Gamma\mathbf{x}_j - cd_i(\mathbf{x}_i - \bar{\mathbf{x}}), \quad i = 1, 2, ..., N, \tag{1}$$

where $\mathbf{x}_i = (x_{i1}, x_{i2}, ..., x_{in})'$, c, $\Gamma \in R^{n\times n}$ and a_{ij} are the state variables of node i, the coupling strength($c > 0$), a matrix linking coupled variables and the elements of the adjacent matrix A, respectively. For the matrix A, if there is an edge between node i and $j(i \neq j)$, then $a_{ij} = a_{ji} = 1$; otherwise $a_{ij} = a_{ji} = 0$. The element a_{ij} $(i = j)$ on the diagonal are $a_{ij} = -k_i$ with k_i the degree of node i.

Suppose controlling all nodes to a homogeneous state $\mathbf{x}_1 = \mathbf{x}_2 = ... = \mathbf{x}_N = \bar{\mathbf{x}} = \mathbf{s}(t)$, $\mathbf{s}(t)$ should satisfy [1, 2]

$$\frac{d\mathbf{s}(t)}{dt} = f(\mathbf{s}(t)). \tag{2}$$

Similar to the master stability function formalism, the stability problem of the system is equivalent to N independent linear systems[1, 4]:

$$\dot{\eta}_k = [\partial f(\bar{\mathbf{x}}) + c\lambda_k\Gamma]\eta_k, \tag{3}$$

where $\partial f(\bar{\mathbf{x}})$ is the Jacobian of f on $\bar{\mathbf{x}}$. λ_k $(k = 1, 2, ..., N)$ are the eigenvalues of matrix B, with

$$B = A - D, \quad D = diag\{d_1, d_2, .., d_N\}, \tag{4}$$

where d_i are the feedback gain of node i. If node i has no feedback gain, d_i=0.

Note that, the stability of equation 3 is simplified as

$$\dot{\eta} = [\partial f(\bar{\mathbf{x}}) + a\Gamma]\eta, \tag{5}$$

Equation 1 can be stabilized to $\mathbf{s}(t)$ on condition that the matrix $[\partial f(\bar{\mathbf{x}}) + a\Gamma]$ is Hurwitz matrix [4], which requires that the real part of $[\partial f(\bar{\mathbf{x}}) + a\Gamma]$ is negative:

$$\mathbf{Re}\{\partial f(\bar{\mathbf{x}}) + a\Gamma\} < 0, \tag{6}$$

where a manifold region S exists that equation 6 is negative if $a \in \mathbf{S}$; otherwise, the system is chaos.

According to the characteristic difference of $\mathbf{S}$, the controlled systems could be divided into three classes[2, 5, 6]:

(1). $\mathbf{S} = \mathbf{S}_1 = (-\infty, \alpha_1)$, where $\alpha_1 \leq 0$. The stable condition is

$$c\lambda_k < \alpha_1, k = 1, 2, ..., N. \tag{7}$$

Since $0 > \lambda_1 \geq \lambda_2 \geq ... \geq \lambda_N$, equation 7 is equivalent to

$$c > \frac{\alpha_1}{\lambda_1}. \tag{8}$$

(2). $\mathbf{S} = \mathbf{S}_2 = (\alpha_2, \alpha_1)$, where $\alpha_2 < \alpha_1 < 0$. The stable condition is

$$c\lambda_N > \alpha_2, \qquad c\lambda_1 < \alpha_1 \tag{9}$$

Since $0 > \lambda_1 \geq \lambda_N$, equation 9 is equivalent to

$$\frac{\alpha_1}{\lambda_1} < c < \frac{\alpha_2}{\lambda_N}. \tag{10}$$

$\frac{\lambda_N}{\lambda_1}$ is a more general metric to characterize the stability:

$$R = \frac{\lambda_N}{\lambda_1} < \frac{\alpha_2}{\alpha_1}. \tag{11}$$

Since lower R represents broader coupling range of coupling strength, lower R is better.

(3). $\mathbf{S} = \mathbf{S}_3 = \Phi$. The system can't be controlled under this condition.

II. SI NOTES: SPEED OF CONTROLLABILITY

The speed of controllability characterizes the rate of convergence in pinning control. To investigate the speed of controllability, a precondition exists that the system is stable. Under this condition, the convergence speed of equation 3 is determined by the largest eigenvalue of $[\partial f(\bar{\mathbf{x}}) + c\lambda_k\Gamma]$.

$$v = \{max(v_k)|v_k = \lambda_1(\partial f(\bar{\mathbf{x}}) + c\lambda_k\Gamma), k = 1, 2, .., N\}. \tag{12}$$

Equation 12 is a general metric to evaluate the speed of controllability. The aim is selecting appropriate pinning nodes to minimize the largest eigenvalue of $[\partial f(\bar{\mathbf{x}}) + c\lambda_k \Gamma]$, i.e., minimize v.

If the stable region is $\mathbf{S} = \mathbf{S}_2 = (\alpha_2, \alpha_1)$, conventional method usually use Eq. 11 to characterize the strength of stability. Previous research mostly focuses on minimizing R to extend the coupling interval (σ_1, σ_2) of c [1, 2]. Low R requires large λ_N, but small λ_1. Smaller R usually indicates larger stable range and better stability. However the minimal R don't always represent minimal v of Eq. 12. For example, we suppose that $\lambda_N = Const.$ As λ_1 decreases, R decreases as a result. But the speed v depends on not only $\lambda_1([\partial f(\bar{\mathbf{x}}) + c\lambda_1 \Gamma])$, but also $\lambda_1([\partial f(\bar{\mathbf{x}}) + c\lambda_N \Gamma])$. So the speed of controllability v may not change. Thus R is not enough to evaluate the control speed of the system when $\mathbf{S} = \mathbf{S}_2$, and previous research about enhancing stability is not suitable for the speed of controllability. Only Equation 12 is appropriate for evaluating the speed of controllability.

In our paper, we suppose $f(\bar{\mathbf{x}}) = 0$ and Γ are usually positive definite($\Gamma > 0$) [3, 4]. Suppose a constant $\rho \quad (\rho < 0)$ exists, such that

$$\partial f(\bar{\mathbf{x}}) + \rho \Gamma < 0. \tag{13}$$

If $c\lambda_k < \rho \quad (\forall i, i = 1, ..., N)$, we can ensure that $\partial f(\bar{\mathbf{x}}) + c\lambda_k \Gamma$ are Hurwitz matrix and the controlled network is exponentially stable[4]. so the stable condition is

$$c \geq c_{min} = |\frac{\rho}{\lambda_1(B)}|, \tag{14}$$

where ρ is a constant. The stable range of c is $c \in (c_{min}, +\infty)$.

Under the stable condition, the speed are mainly determined by the largest eigenvalue of $\lambda_1(B)$, Equation 12 is simplified as

$$v = \lambda_1([\partial f(\bar{\mathbf{x}}) + c\lambda_1 \Gamma]). \tag{15}$$

lower $\lambda_1(B)$ represents lower negative eigenvalues of $[\partial f(\bar{\mathbf{x}}) + c\lambda_1 \Gamma]$ and higher speed of controllability. Thus, $\lambda_1(B)$ is also utilized to characterize the speed of controllability for a network in the paper.

III. SI NOTES: INFLUENCE OF RESTRICTION E_{sum}

Figure S1 depicts $\lambda_1(\bar{A})$ as a function of δ and C at $\alpha = -0.6$. The proposed approach has a similar performance with that of large-degree selection in BA model network. However, it

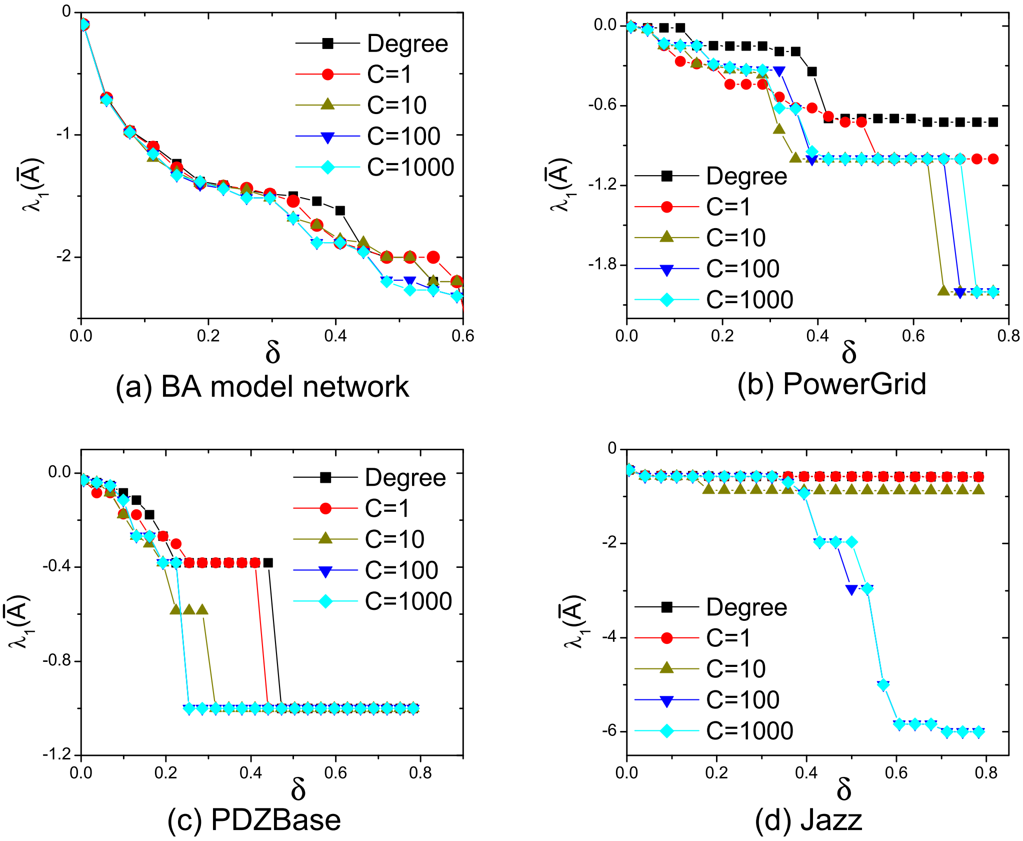

FIG. S1 (Color online) The largest eigenvalue $\lambda_1(\bar{A})$ as a function of δ and C for four networks at $\alpha = -0.6$. In large-degree pinning control, the pinning nodes are obtained by selecting the largest $\lfloor N\delta \rfloor$ degree nodes. The results show that the proposed approach can efficiently enhance the speed of pinning controllability when C is properly large.

performs better than degree pinning control in other three real networks and the differences are due to the special topologies of real networks. Note that, the only difference is the lines $C = 1$ in Fig. S1(b) and S1(d), and these lines have similar performance with those of large-degree pinning control. When C is small, restricted by $E_{sum} = C = 1$, only some high degree nodes obtain high feedback gains and are selected as pinning nodes. Therefore, both approaches have similar speed of pinning controllability. In practice, we can increase properly C to avoid the trap of performance.

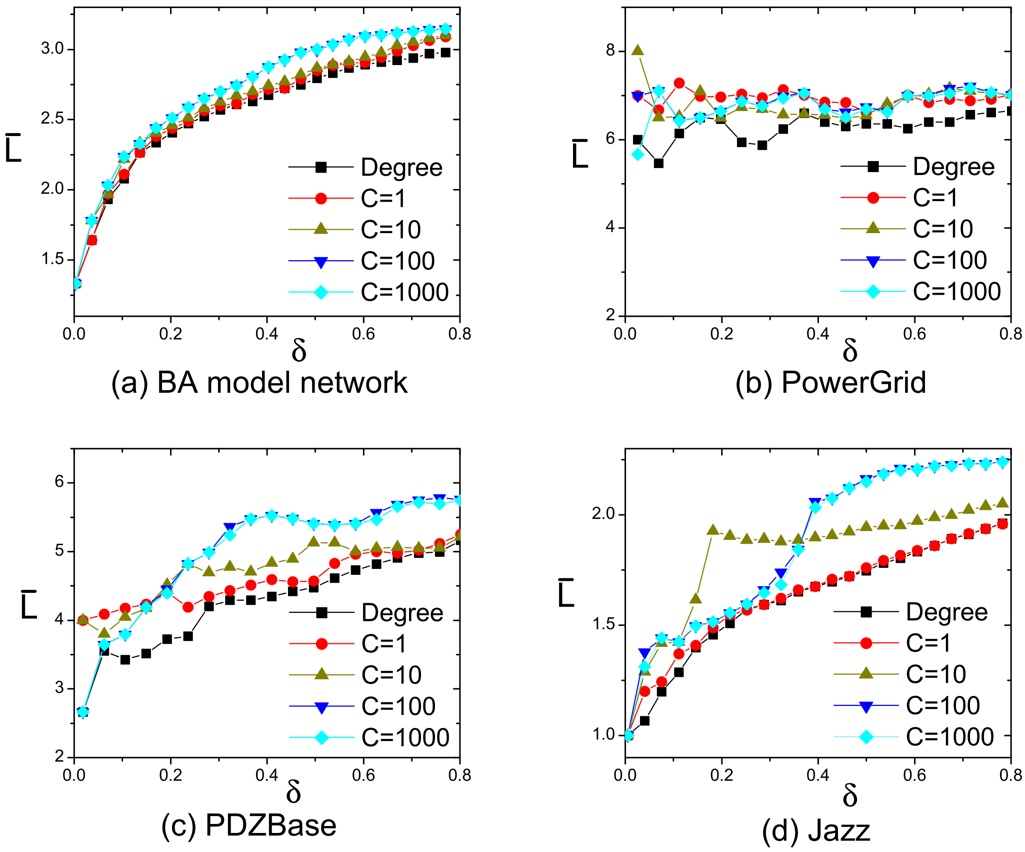

FIG. S2 (Color online) The average distance $\bar{L}$ as a function of δ and C for four networks at $\alpha = -0.6$. In large-degree pinning control, the pinning nodes are obtained by selecting the largest $\lfloor N\delta \rfloor$ degree nodes.

IV. SI NOTES: SPEED OF CONTROLLABILITY BASED ON BETWEENNESS

Since network topology has a great influence on the controllability [7] and the importance of a node relates much to the structure of the network [8], apart from degree of nodes, the importance of a node could also be evaluated by its betweenness [9, 10]. A node's betweenness is defined as the number of shortest paths from all vertices to all others that pass through that node. A node with high betweenness has a large influence on the transfer of information through the network, under the assumption that information transfer follows the shortest paths. Here, we suppose that $w_i = g_i$, where g_i is the betweenness of node i.

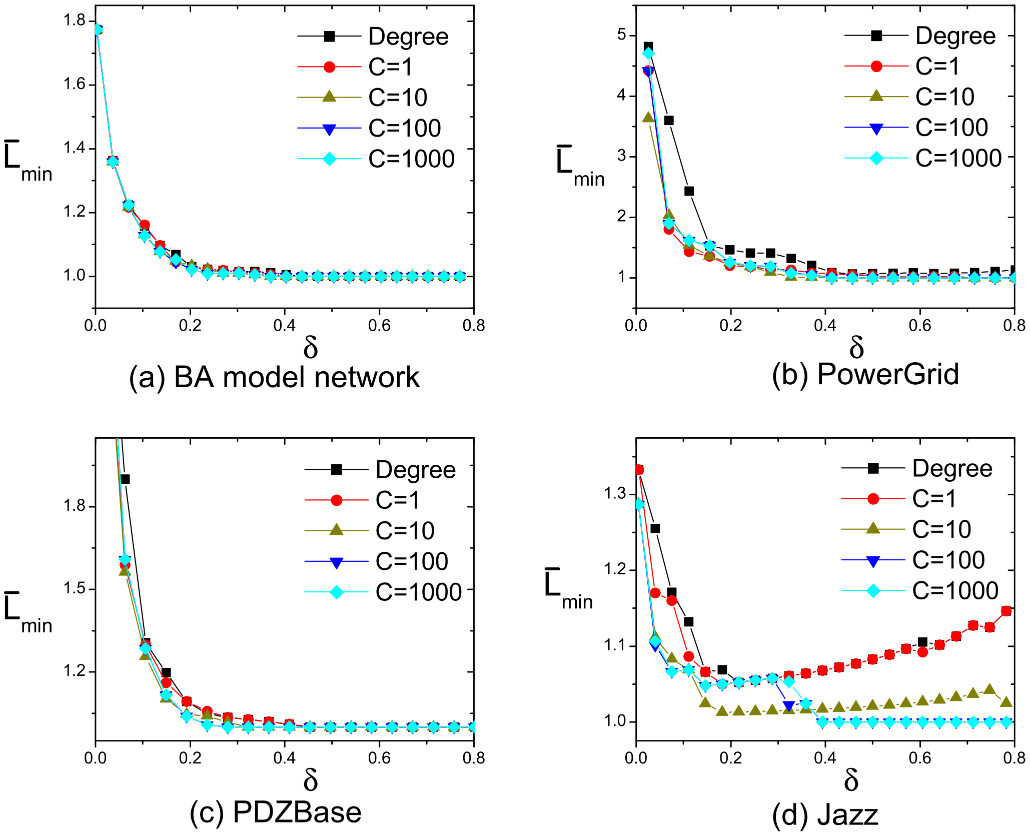

FIG. S3 (Color online) The average shortest distance $\bar{L}_{min}$ as a function of δ and C for four networks at $\alpha = -0.6$. In large-degree pinning control, the pinning nodes are obtained by selecting the largest $\lfloor N\delta \rfloor$ degree nodes.

The results are shown in Fig. S4-S11.

In Figure S4 and Figure S5, the results are similar to that of $w_i = k_i$. The proposed method also enhances the speed of controllability a lot compared to betweenness control in Fig. S6 and Fig. S7. The reason is that proposed method selects sparser pinning nodes than that of betweenness selection(See Fig. S8 and Fig. S9). However, the performances of $\bar{L}_{min}$ are almost the same for both methods(See Fig. S10 and Fig. S11), which are due to the boundary restriction of $\bar{L}_{min}(\bar{L}_{min} >= 1)$. Both methods arrive at the $\bar{L}_{min} = 1$. Though $\bar{L}_{min}$ of both methods have similar performance, the sparsity could be distinguished by Fig. S8 and Fig. S9.

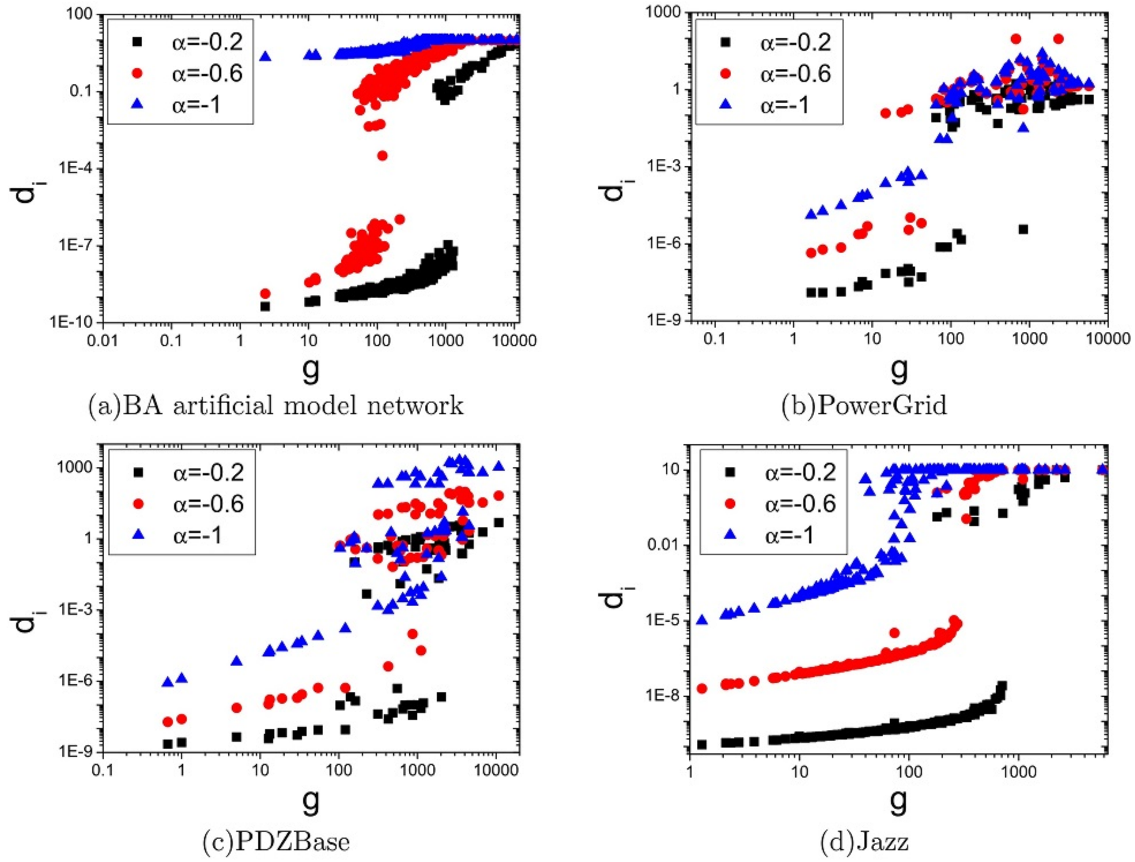

(a)BA artificial model network (b)PowerGrid

(c)PDZBase (d)Jazz

FIG. S4 The feedback distribution of four networks for $\alpha = -0.2, -0.6, -1$ at $C = 10$. The results are obtained by LMI optimization method. The accuracy of λ_x is 1×10^{-6} in the optimization process.

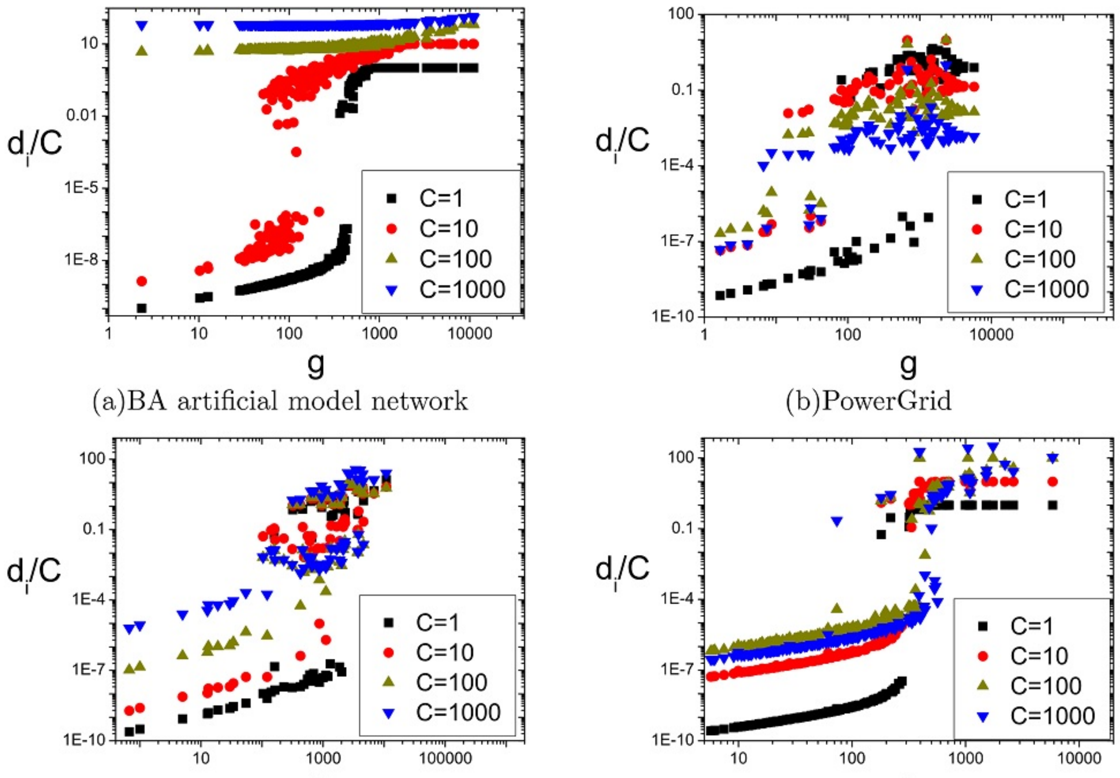

(a)BA artificial model network (b)PowerGrid

(c)PDZBase (d)Jazz

FIG. S5 (Color online) The feedback distribution of four networks for $C = 1, 10, 100, 100$ at $\alpha = -0.6$. The results are obtained by LMI optimization method and the accuracy of λ_x is 1×10^{-6} in the optimization process.

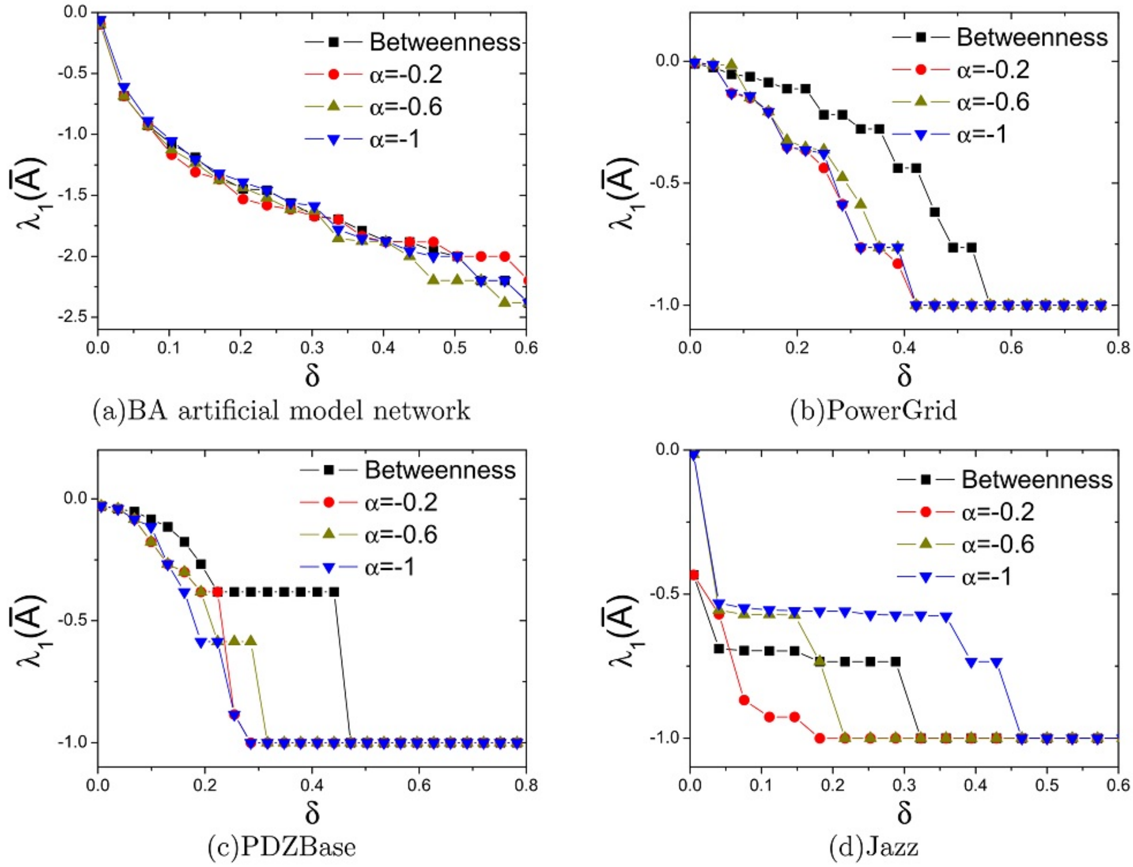

FIG. S6 (Color online) The largest eigenvalue $\lambda_1(\bar{A})$ for an artificial network and three practical networks as a function of δ and α at $C = 10$. In betweenness pinning control, the pinning nodes are obtained by selecting the largest $\lfloor N\delta \rfloor$ betweenness nodes.

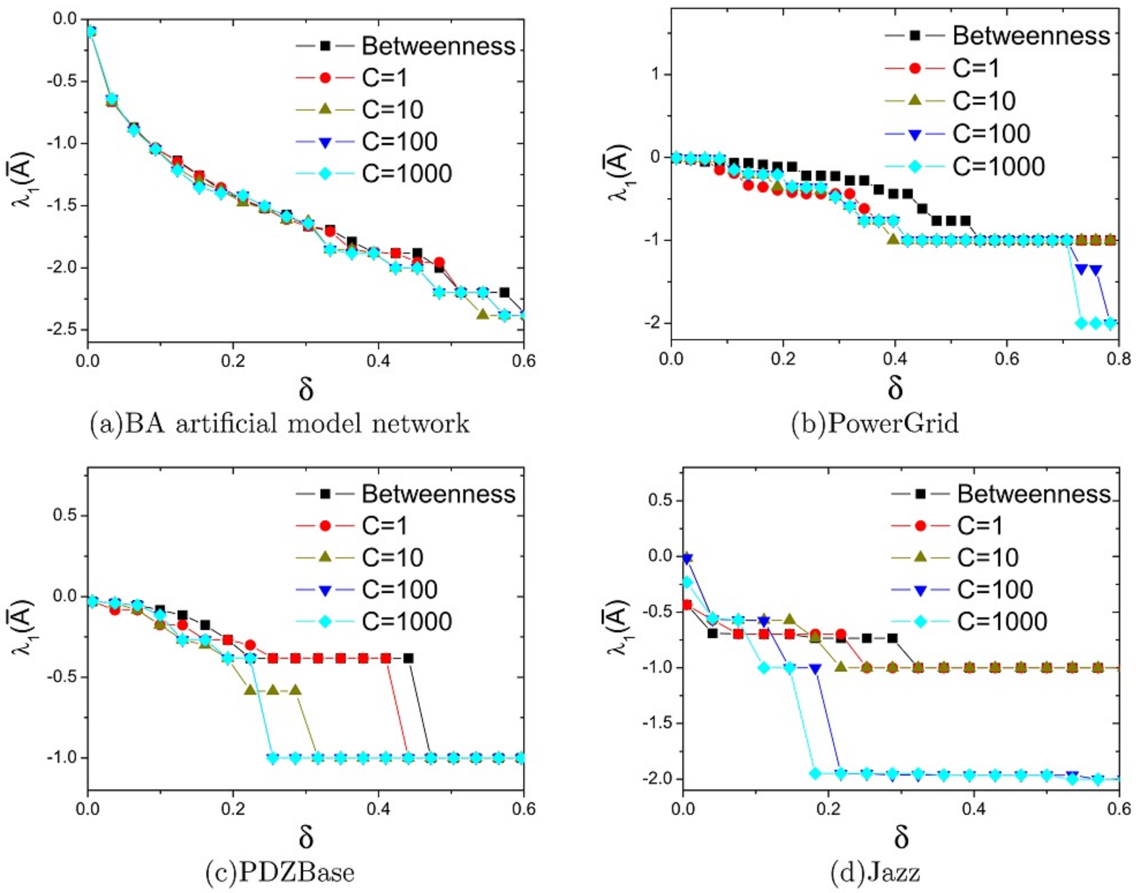

FIG. S7 (Color online) The largest eigenvalue $\lambda_1(\bar{A})$ for an artificial network and three practical networks as a function of δ and C at $\alpha = -0.6$. In betweenness pinning control, the pinning nodes are obtained by selecting the largest $\lfloor N\delta \rfloor$ betweenness nodes.

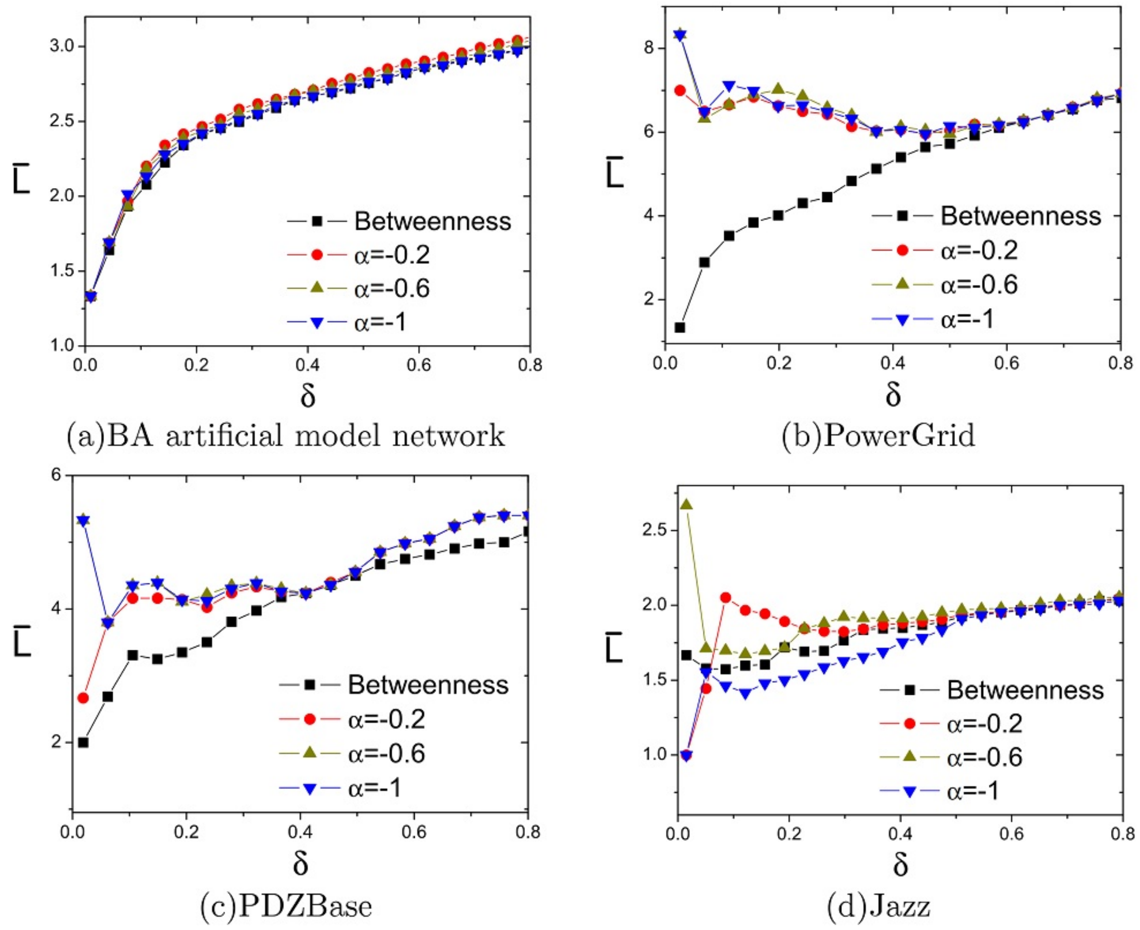

FIG. S8 (Color online) The average distance $\bar{L}$ as a function of δ and α at $C = 10$.

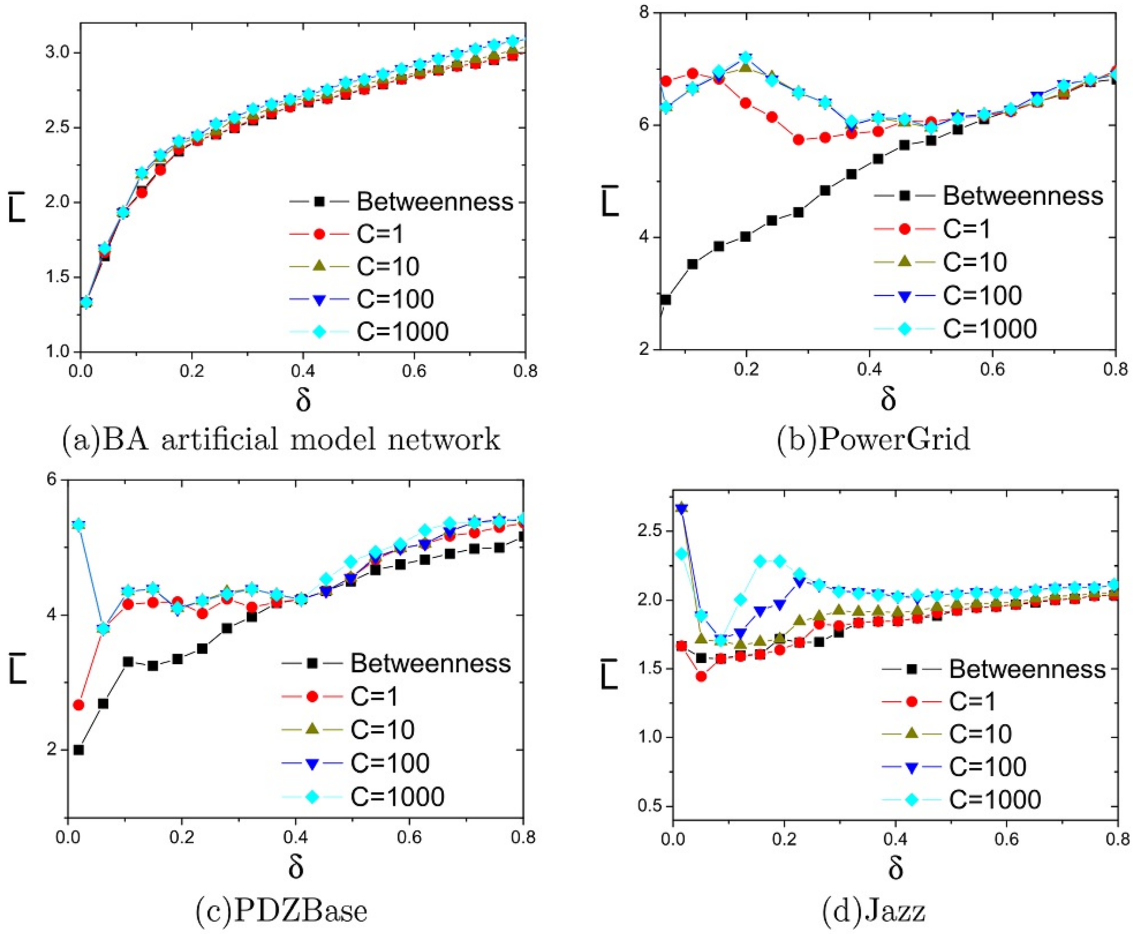

FIG. S9 (Color online) The average distance $\bar{L}$ as a function of δ and C at $\alpha = -0.6$.

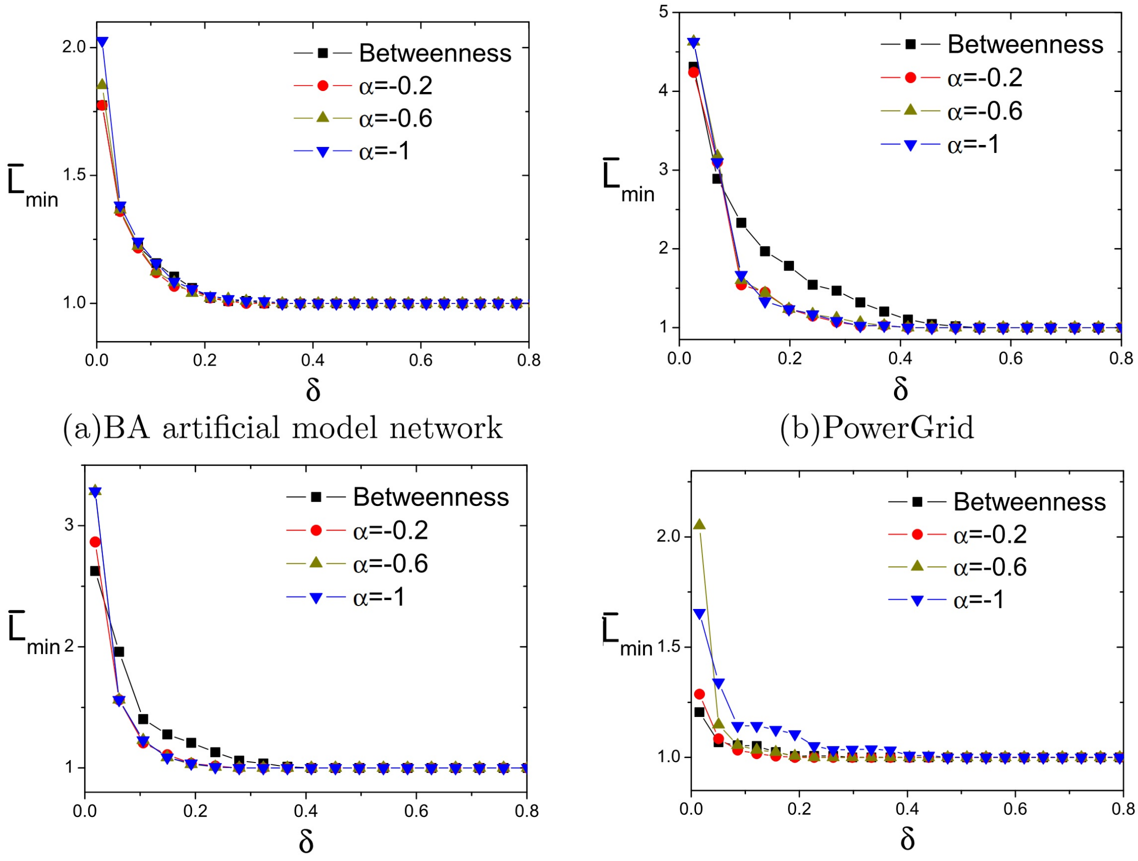

(a)BA artificial model network (b)PowerGrid

(c)PDZBase (d)Jazz

FIG. S10 (Color online) The average shortest distance $\bar{L}_{min}$ as a function of δ and α at $C = 10$.

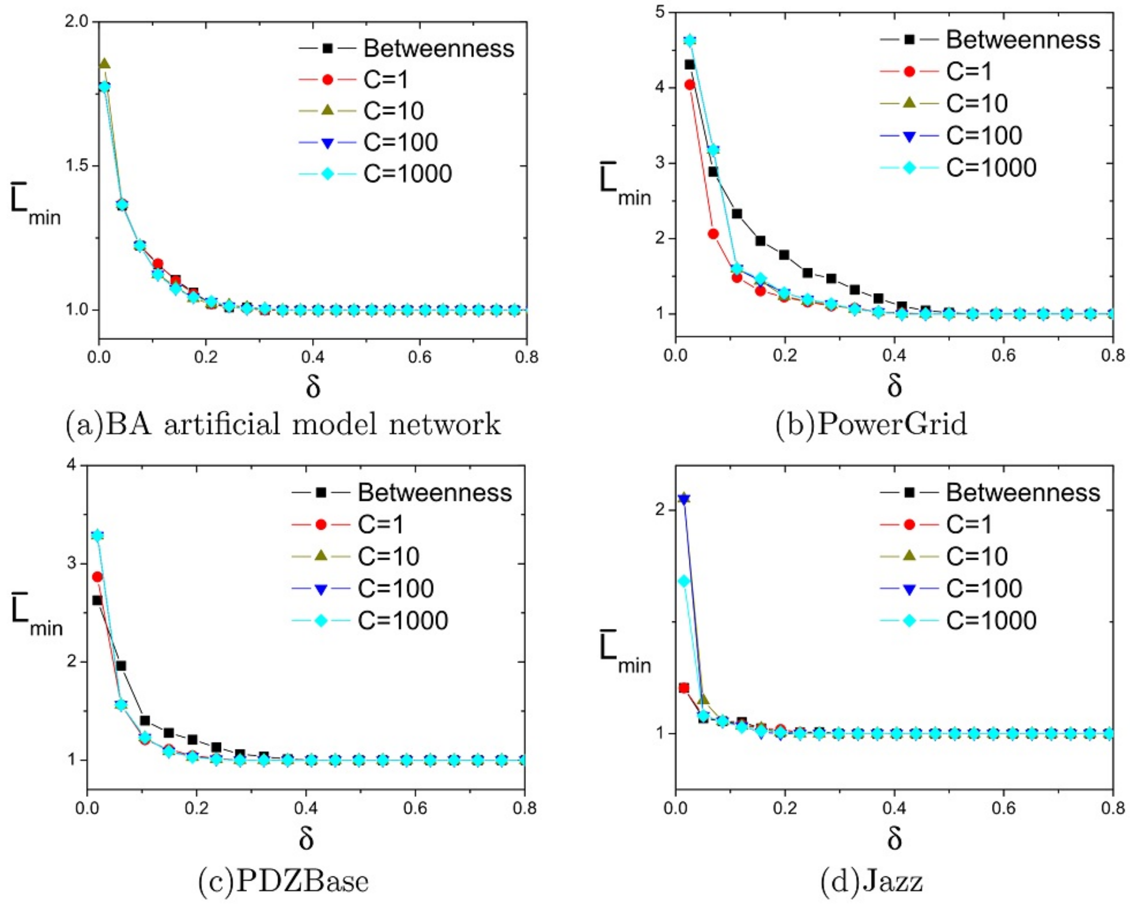

(a)BA artificial model network

(b)PowerGrid

(c)PDZBase

(d)Jazz

FIG. S11 (Color online) The average shortest distance $\bar{L}_{min}$ as a function of δ and C at $\alpha = -0.6$.

[1] Jalili, M., Sichani, O. A. & Yu, X. Optimal pinning controllability of complex networks: Dependence on network structure. *Phys. Rev. E* **91**, 012803 (2015).

[2] Sorrentino, F., di Bernardo, M., Garofalo, F. & Chen, G. Controllability of complex networks via pinning. *Phys. Rev. E* **75**, 046103 (2007).

[3] Wang, X., Li, X. & Lu, J. Control and flocking of networked systems via pinning. *IEEE Circuits Syst. Mag.* **10**, 83–91 (2010).

[4] Wang, X. F. & Chen, G. Pinning control of scale-free dynamical networks. *Physica A* **310**, 521–531 (2002).

[5] Kocarev, L. & Amato, P. Synchronization in power-law networks. *Chaos* **15**, 024101 (2005).

[6] Pecora, L. M. Synchronization conditions and desynchronizing patterns in coupled limit-cycle and chaotic systems. *Phys. Rev. E* **58**, 347 (1998).

[7] Wu, C. W. On the relationship between pinning control effectiveness and graph topology in complex networks of dynamical systems. *Chaos* **18**, 037103 (2008).

[8] Tang, Y., Gao, H., Zou, W. & Kurths, J. Identifying controlling nodes in neuronal networks in different scales. *PloS ONE* **7**, e41375 (2012).

[9] Mahadevan, P. *et al.* Lessons from three views of the Internet topology. *arXiv preprint cs/0508033* (2005).

[10] Barthelemy, M. Betweenness centrality in large complex networks. *Eur. J. Phys. B* **38**, 163–168 (2004).
